# A fatal case of *COQ7*‐associated primary coenzyme Q_10_ deficiency

**DOI:** 10.1002/jmd2.12032

**Published:** 2019-04-03

**Authors:** Anna K.‐Y. Kwong, Annie T.‐G. Chiu, Mandy H.‐Y. Tsang, Kin‐Shing Lun, Richard J. T. Rodenburg, Jan Smeitink, Brian H.‐Y. Chung, Cheuk‐Wing Fung

**Affiliations:** ^1^ Department of Paediatrics and Adolescent Medicine, Li Ka Shing Faculty of Medicine Queen Mary Hospital, The University of Hong Kong Hong Kong SAR China; ^2^ Radboud Centre for Mitochondrial Medicine, Department of Paediatrics, Radboud Institute for Molecular Life Sciences Radboud University Nijmegen Medical Centre Nijmegen The Netherlands

**Keywords:** coenzyme Q_10_, CoQ_10_, CoQ_10_ supplementation, *COQ7*, encephalo‐myo‐nephro‐cardiopathy, mitochondrial disease

## Abstract

**Background:**

Primary coenzyme Q_10_ (CoQ_10_) deficiencies are clinically and genetically heterogeneous group of disorders associated with defects of genes involved in the CoQ_10_ biosynthesis pathway. *COQ7*‐associated CoQ_10_ deficiency is very rare and only two cases have been reported.

**Methods and Results:**

We report a patient with encephalo‐myo‐nephro‐cardiopathy, persistent lactic acidosis, and basal ganglia lesions resulting in early infantile death. Using whole exome sequencing, we identified compound heterozygous variants in the *COQ7* gene consisting of a deletion insertion resulting in frameshift [c.599_600delinsTAATGCATC, p.(Lys200Ilefs*56)] and a missense substitution [c.319C>T, p.(Arg107Trp), NM_016138.4]. Skin fibroblast studies showed decreased combined complex II + III activity and reduction in CoQ_10_ level.

**Conclusion:**

This third patient presenting with lethal encephalo‐myo‐nephro‐cardiopathy represents the severe end of this ultra‐rare mitochondrial disease caused by biallelic *COQ7* mutations. The response to CoQ_10_ supplement is poor and alternative treatment strategies should be developed for a more effective management of this disorder.

## 1 INTRODUCTION

Coenzyme Q_10_ (CoQ_10_), known as ubiquinone, serves as a mitochondrial respiratory chain electron carrier shuttling electrons from NADH:ubiquinone oxidoreductase (complex I) or succinate dehydrogenase (complex II) to ubiquinol cytochrome *c* reductase (complex III) in the inner mitochondrial membrane.^1^ Besides, CoQ_10_ functions as an antioxidant to provide protection against lipid peroxidation as well as DNA and protein oxidation in animal cells.^1^ CoQ_10_ consists of a polar benzoquinone ring for redox reaction and a hydrophobic isoprenyl tail for diffusion in lipid bilayer and interaction with redox enzymes.^2^ In eukaryotes, at least 16 enzymes have been identified or proposed for CoQ10_10_ biosynthesis.^3^

Primary CoQ_10_ deficiency is clinically and genetically heterogeneous with an extremely wide spectrum of clinical manifestations. A recent review classified the phenotypes according to the genetic defects of the CoQ_10_ biosynthesis pathway: (a) glomerular renal involvement manifested as steroid resistant nephrotic syndrome (SRNS) associated with the defects of *PDSS2* (COQ1 subunit 2), *COQ2*, *COQ6*, or *COQ8B*; (b) encephalomyopathy with *COQ4*, *COQ7*, or *COQ9* defects involving hypertrophic cardiomyopathy, lactic acidosis and tubulopathy; and (c) predominant cerebellar ataxia involving only *COQ8A*.^4^ The genes associated with CoQ_10_ deficiency were suggested to have additional roles in mitochondrial homeostasis in addition to CoQ_10_ biosynthesis, which may account for the clinical heterogeneity.^4^ The biological mechanisms regulating various clinical phenotypes associated with different genetic defects are still unclear.

Primary CoQ_10_ deficiency caused by defect of the *COQ7* gene is the most rarely reported. *COQ7* encodes for 5‐demethoxyubiquinone hydroxylase that catalyzes the hydroxylation of 2‐polyprenyl‐3‐methyl‐6‐methoxy‐1,4‐benzoquinol (DMQH_2_), a critical step in CoQ_10_ biosynthesis. In addition to CoQ_10_ biosynthesis, a previous study identified a nuclear form of COQ7 which was increased in response to reactive oxygen species (ROS) and functioned independently to regulate ROS metabolism, stress responses and longevity.^5^ The first case reported was a 9‐year‐old Syrian boy with mildly progressive encephalo‐neuro‐nephro‐cardiopathy which was stabilized by CoQ_10_ treatment.^6^ The second case reported recently was a 6‐year‐old girl presented with spasticity and bilateral sensorineural hearing loss.^7^ Here, we report a Chinese boy with compound heterozygous *COQ7* variants, presenting with a phenotype of mitochondrial encephalo‐myo‐nephro‐cardiopathy, persistent lactic acidosis, basal ganglia lesions and decrease in CoQ_10_ level in the skin fibroblasts. This is the third reported case of *COQ7* defect associated with primary CoQ_10_ deficiency. The discovery of the present case has led to a wider clinical phenotypic spectrum of *COQ7*‐associated CoQ_10_ deficiency ranging from spasticity or mildly progressive encephalo‐neuro‐nephro‐cardiopathy to a fatal multisystemic disorder.

## 2 CASE REPORT

### 2.1 Clinical history

The index case was born of a nonconsanguineous Chinese couple as the second twin of a dichorionic diamniotic twin pregnancy. The first twin was healthy and unaffected. Our index patient was noted to have intrauterine growth restriction, cardiomegaly and tricuspid regurgitation since antenatal period. He was born at 33 weeks of gestation by emergency cesarean section due to oligohydramnios and abnormal Doppler signals. His birth weight was 1.6 kg (10th percentile) with satisfactory Apgar scores (Table 1). Surfactant was given shortly after birth.

**Table 1:** Clinical features of the three cases of *COQ7* pathogenic variants reported in literature

	Freyer et al	Wang et al	Index patient
Ancestry	Syrian	–	Chinese
Parents	Consanguineous	Consanguineous	Nonconsanguineous
Antenatal	Oligohydramnios, fetal lung hypoplasia, growth retardation	Gestational diabetes	Oligohydramnios, growth retardation, fetal cardiomegaly
Gestational age	Full term	37 wk	33 wk
Respiratory	Lung hypoplasia with persistent pulmonary hypertension of newborn	–	Central hypoventilation
Renal	Renal dysfunction with small dysplastic kidneys with impaired cortical differentiation (resolved upon follow‐up)	–	Multiple renal cysts and diffuse increase in renal parenchymal echogenicity with accentuation of cortico‐medullary differentiation
Cardiovascular	Left ventricular cardiac hypertrophy (with subsequent regression), systemic hypertension	–	Severe hypertrophic cardiomyopathy with pericardial effusion, moderate tricuspid regurgitation
Growth and feeding	Postnatal growth retardation with oromotor dysfunction requiring gastrostomy	Normal	Postnatal growth retardation with oromotor dysfunction requiring tube feeding
Neurology and developmental outcome	Normal MRI brain	Normal MRI brain	MRI brain showed subdural hematoma, basal ganglial and thalami hypodensities with abnormal lactate peak
	Distal contractures since birth with progressive peripheral sensorimotor polyneuropathy, axonal, and demyelinating type	Generalized muscle wasting, more prominent in the legs, also affecting temporalis muscle	Generalized hypotonia with progressive myopathy clinically
	Mild learning difficulties at 9 y old, never learned to stand or walk independently	Normal early developmental milestones, followed by language delay since 14 mo, progressive motor regression since second year and became wheelchair bound at 3	Global developmental delay with developmental age below 3 mo across all domains at 1 y
Hearing	Combined sensorineural and conduction hearing impairment	Bilateral low frequency sensorineural hearing loss	Bilateral profound hearing impairment in range of 2‐4 Hz
Vision	Visual dysfunction	–	Lack of visual following
Respiratory chain enzyme activities	Complex I + III and IV deficiency	–	Complex II + III deficiency with normal isolated activity of complex II and complex III
*COQ7* variants identified	p.(Val141Glu)	p.(Leu111Pro)	p.(Lys200Ilefs*56), p.(Arg107Trp)
Response to treatment	Dosage of CoQ_10_ not available	CoQ_10_ 11.4 mg/kg twice daily	CoQ_10_ 4 mg/kg/day since 2 mo
	Regression stalled	No obvious improvement	Stepped up to 20 mg/kg/day at 1 y
	Significant reduction in neuromuscular pain	No deterioration or worsening spasticity	No obvious response to treatment

Abbreviation: MRI, magnetic resonance imaging; y, years; mo, months.

However, he developed heart failure with respiratory distress since day 4 of life, culminating in a re‐intubation at day 24 of life. Repeated echocardiography at 26 days old showed severe hypertrophic cardiomyopathy with moderately impaired right systolic function, moderate tricuspid regurgitation and pericardial effusion. There was significant bi‐atrial enlargement suggestive of diastolic dysfunction, with mild outflow obstruction at both ventricles. Heart failure symptoms persisted despite optimal medical therapy.

Respiratory‐wise, he was ventilator‐dependent for 7 months due to heart failure, with subsequent extubation to nasal cannula. Since 10 months, he was started on noninvasive ventilation due to central hypoventilation.

**Figure 1:**
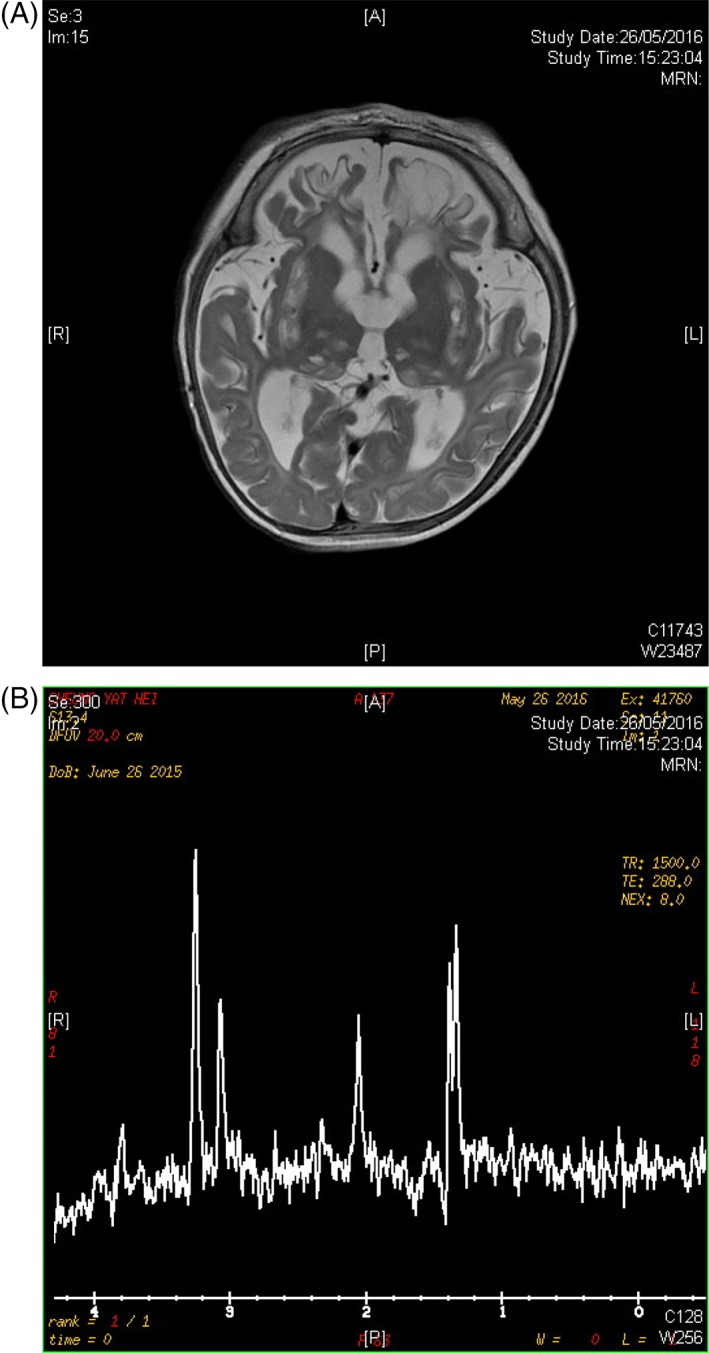
Magnetic resonance imaging brain images of the index subject. A, T2W hyperintense cystic changes involving bilateral corona radiata, basal ganglia and thalami, compatible with old lacunar infarcts, cerebral atrophy with encephalomalacic changes in bilateral frontal lobes. B, Doublet lactate peaks on magnetic resonance spectroscopy Abbreviation: MRI, magnetic resonance imaging

Neurologically, he was noted to have generalized hypotonia, ptosis, bilateral severe visual impairment, profound hearing impairment with progressive loss of muscle bulk and muscle weakness especially over bilateral lower limbs despite preserved jerks. Soft dysmorphic features were also noted with frontal bossing, low nasal bridge and sparse hair. He also developed infantile spasms since 10 months of life, which were responsive to phenobarbitone and vigabatrin. Magnetic resonance imaging (MRI) of the brain at 10 months of age showed multiple T2W hyperintense cystic changes involving bilateral corona radiata, basal ganglia and thalami, compatible with old lacunar infarcts, cerebral atrophy with encephalomalacic changes in bilateral frontal lobes, features of periventricular leukomalacia as well as doublet lactate peaks on magnetic resonance spectroscopy (see Figure 1A and B).

Ultrasound of the urinary system showed multiple renal cysts and a diffuse increase in renal parenchymal echogenicity with accentuation of cortico‐medullary differentiation. Renal function was unremarkable.

Metabolic workup showed persistently elevated lactate up to 17 mmol/L with a raised lactate/pyruvate ratio of 50. His alanine level was up to 463 μmol/L (reference: 143‐439 μmol/L). Urine for organic acids showed increased lactate, pyruvate, 3‐hydroxybutyrate, dicarboxylic aciduria and increased excretions of Kreb cycle intermediates. These, together with the neuroimaging findings were highly suggestive of a mitochondrial disorder. He was started on CoQ_10_ at 2 months of life and the dose was further stepped up to 20 mg/kg/day at 12 months of life. Around the same time, he had progressive cardiorespiratory deterioration and was eventually succumbed during an episode of deterioration due to sepsis.

### 2.2 Genetic analysis

Whole exome sequencing (WES) and bioinformatics analyses were performed as described previously.^8^, ^9^ The variants called were annotated by Oncotator version 1.8.0.0, filtered and selected using gene panels associated with mitochondrial diseases and with strong support of mitochondrial localization suggested in MitoCarta 2.0. Compound heterozygous variants were identified in the *COQ7* gene. The variants were further confirmed by Sanger sequencing. One of the variants was a deletion insertion resulting in frameshift [c.599_600delinsTAATGCATC, p.(Lys200Ilefs*56), NM_016138.4] in exon 6 which formed the stop codon 37 amino acid downstream to the wildtype stop codon. Another variant was a missense one [c.319C>T, p.(Arg107Trp), NM_016138.4] in exon 3. Both variants were not found in East Asian population according to The Genome Aggregation Database (gnomAD).

The amino acid residue of p.(Arg107Trp) is located in a highly evolutionary conserved region by multiple alignments of 100 vertebrates shown in UCSC Genome Browser with a high GERP score of 5.91 for mammalian alignment. In silico analysis including SIFT, Polyphen‐2, and Mutation Taster also predicted that the residue was located in highly conserved region and p.(Arg107Trp) was predicted to be damaging to the protein structure and function. Using the 3D modeling by STRUM, the p.(Arg107Trp) variant results a delta‐delta G value of 0.57. A positive delta‐delta G value implies the variant is responsible for COQ7 protein fold stabilization.^10^

Sanger sequencing of the identified variants in parental DNA was performed and showed that the variants were segregated between the parents. Father is the carrier of frameshift variant and mother is the carrier of missense variant. According to ACMG classification,^11^ the frameshift variant falls into the tier of “Likely pathogenic.” It was predicted to undergo nonsense mediated decay with the exon present in the biologically‐relevant transcript.^12^ The missense variant falls into the tier of “Uncertain significance” with a Post_P value 0.5 using the Bayesian classification framework.^13^

### 2.3 Reverse transcription polymerase chain reaction

RNA was extracted from patient's fibroblasts and semi‐quantitative reverse transcription polymerase chain reaction was performed to amplify the *COQ7* complementary DNA (cDNA) consisting of the region with the two variants found. Sanger sequencing of the cDNA revealed that only the allele with the missense variant was expressed while cDNA with the frameshift variant was not identified. This finding illustrated that the mRNA with the frameshift variant could be eliminated by nonstop decay pathway which targets transcripts that do not have an in‐frame stop codon.^14^

### 2.4 Respiratory chain enzymologies analysis

Measurement of the respiratory chain succinate: cytochrome *c* oxidoreductase activity in skin fibroblasts revealed a significant decrease of combined complex II + III (114 mU/UCOX; reference: 325‐649) while the isolated activity of complex II and complex III were within the normal range. Fibroblast CoQ_10_ quantification showed a reduced level to 0.29 nmol/UCOX (reference: 1.64‐3.32). The result of these analyses indicated that this patient has CoQ_10_ deficiency.

## 3 DISCUSSION

### 3.1 Third reported case of pathogenic variant in COQ7

In this study, we reported the third case of *COQ7* defect associated with primary CoQ_10_ deficiency through WES. This again demonstrated that WES is an important molecular diagnostic test for mitochondrial disorders as a similar clinical phenotype (such as multiple organ involvement) can be resulted from mutations of different mitochondrial or nuclear genes.^9^ We have chosen skin biopsy as a less invasive investigation for complex activity and CoQ_10_ level because a previous study illustrated that analysis of fibroblasts was useful to demonstrate a deficiency even though the muscle showed false negative results.^15^ However, a normal CoQ_10_ level in fibroblasts did not exclude CoQ_10_ deficiency in some cases.^16^, ^17^ Tissue specificity suggested that the choice of appropriate tissues is important and study of different tissues in case of a negative result is necessary to confirm CoQ_10_ deficiency.

### 3.2 Comparison with previous cases of COQ7 and COQ9 deficiency

Our subject presented with encephalo‐myo‐nephro‐cardiopathy, persistent lactic acidosis and basal ganglia lesions. The clinical presentation of our patient was comparable to one of reported classical phenotypes of CoQ_10_ deficiency as encephalomyopathy with hypertrophic cardiomyopathy, lactic acidosis and tubulopathy^4^ and this verified that COQ7 deficiency could cause multiple organ involvement. There were many similarities between our index subject and that of the previously reported cases of COQ7 deficiency. All three subjects had hearing impairment and global developmental delay or intellectual disability, with involvement of the peripheral nervous system. Despite the similarities, there were also many notable differences among the three cases. Our index subject had a fatally progressive multisystemic involvement with cardiac failure and clinically significant tubulopathy. The MRI brain showed typical features of a mitochondrial disorder which were absent in the previously reported cases. What remains to be better ascertained, is the response to CoQ_10_ supplements. A comparison of the three patients is available in Table 1.

Different types and positions of the *COQ7* variants could alter the function of COQ7 protein in different ways and affect the presenting phenotypes of the patients. The second case study demonstrated that the variant p.(Leu111Pro) showed a milder reduction in CoQ_10_ level than the p.(Val141Glu) variant in the first case and so the phenotype of the second case is milder.^7^ The p.(Val141Glu) variant next to p.Glu142 residue is predicted to be part of the di‐iron motif and was suggested to impair iron binding for hydroxylation.^18^ The p.(Arg107Trp) variant in the present study was located in another highly conserved region but the biological significance of that region is not well‐studied. The 3D modeling by STRUM implies that the p.(Arg107Trp) variant is responsible for COQ7 protein fold stabilization.^10^ Further, mutations at the COQ7 Arg107 residue is predicted to affect the interaction with COQ9, disrupting the CoQ_10_ biosynthesis.^19^ A change from Arg to Trp results in an increased size, increased in hydrophobicity and a loss in charge, suggesting a loss of interactions with other molecules. Further in‐vitro investigation on COQ7 structure and function can provide more insights to the phenotype‐genotype relationship.

As COQ9 was suggested to interact with COQ7 physically and functionally in CoQ_10_ biosynthesis,^20^ the clinical phenotypes were compared. Up till now, six patients from three families were reported to have COQ9 deficiency.^21^, ^22^, ^23^ Both COQ7 and COQ9 deficiency may cause a multisystemic disorder with encephalopathy, lactic acidosis and a variable combination of cardiopathy and nephropathy. However, patients with biallelic *COQ9* mutations all had severely intractable symptoms causing neonatal or infantile death and a lack of response to CoQ_10_ supplementation, similar to our index patient. Intriguingly, the other two patients with biallelic *COQ7* mutations as mentioned above had a much milder phenotype with variable response to CoQ_10_. Understanding the precise role of COQ9 in CoQ_10_ biosynthesis would be necessary to explain the phenotypic difference between COQ7 and COQ9 deficiency.

### 3.3 Role of CoQ_10_ supplements in mitochondrial disorders

In contrast to the first case with the clinical progression being stabilized by CoQ_10_ treatment, our patient had no response to CoQ_10_ supplementation at a recommended dose of 20 mg/kg/day.^24^ However, a higher dose of 30 mg/kg/day could have been tried.^25^ CoQ_10_ deficiency is potentially treatable by CoQ_10_ supplementation but the outcomes are still variable. Different tissue involvements in different cases may influence the responsiveness to CoQ_10_ treatments. In a previous report, CoQ_10_ supplementation is effective in patient with *COQ2* defect manifested with neurological signs, nephrotic syndrome or stroke‐like episode involving vascular structures.^26^ Besides, improvements including better coordination, decrease in muscle weakness, better speech articulation, etc. were reviewed.^27^ However, the responses of central nervous system are poor for some patients such as Leigh syndrome caused by *PDSS2* defects and refractory seizures resulting from *COQ9* mutations.^22^, ^28^ Other neurological symptoms including developmental delay were also not improved.^27^ Possible contributing factors are poor bioavailability of CoQ_10_ leading to poor penetration across the blood‐brain barrier and presence of irreversible brain damage before CoQ_10_ treatment.^29^ These can be the reasons for poor CoQ_10_ responsiveness in our patient, owing to the early progressive and severe neurological damages.

As demonstrated by this first report,^6^ 2,4‐dihydroxybensoic acid (2,4DHB), a CoQ10 biosynthetic precursor with an additional hydroxyl group which is normally added by COQ7, was able to rescue the CoQ_10_ deficiency in patient fibroblasts by bypassing the need for COQ7 and this finding was supported by another in vivo study.^30^ On the other hand, the second case report demonstrated that 2,4DHB, at the same time, can inhibit native CoQ_10_ biosynthesis.^7^ In *Caenorhabditis elegans* model, overexpression of the *CLD1* gene, encoding a phospholipase A, restored CoQ_10_ wildtype levels, suggesting a recovery of COQ7 function by structural remodeling.^31^ All these studies highlighted nonlinearity of CoQ_10_ biosynthesis and opened up alternative treatment strategies for CoQ_10_ deficiency.

In conclusion, the present study reported a severe case of *COQ7*‐associated primary CoQ_10_ deficiency with progressive and fatal encephalo‐myo‐nephro‐cardiopathy not responding to CoQ_10_ treatment. Alternative treatment strategies should be developed for a more effective management of this disorder.

## CONFLICT OF INTERESTS

A.K.Y.K., A.T.G.C., M.H.Y.T., K.‐S.L., R.J.T.R., B.H.Y.C., and C.W.F. declare that they have no conflict of interest. J.S. is the CEO of Khondrion, a pharmaceutical company developing compounds to potentially treat mitochondrial disease.

## AUTHOR CONTRIBUTIONS

Conception and design of study: A.K.Y.K., C.W.F., and B.H.Y.C. Drafting the manuscript: A.K.Y.K., C.W.F., and A.T.G.C. Evaluation of manuscript for content: A.K.Y.K., C.W.F., B.H.Y.C., T.M.H.Y., K.S.L., and J.S. Data analysis and interpretation: A.K.Y.K., A.T.G.C., T.M.H.Y., and R.J.T.R.

## ETHICAL APPROVAL STATEMENT

Ethical approval had been obtained from the Institutional Review Board (IRB) of the University of Hong Kong‐Hong Kong West Cluster (IRB Ref. No.: UW 11‐190). Written consent was obtained from the parents of the patient.
